# Supplementary material for: MITF Downregulation Induces Death in Human Mast Cell Leukemia Cells and Impairs IgE-Dependent Degranulation
Source: Int J Mol Sci. 2023 Feb 9;24(4):3515. doi: 10.3390/ijms24043515 (PMC9961600; doi:10.3390/ijms24043515)
Supplement: Supplementary file 1 [file ijms-24-03515-s001.zip › Supplementary materials.pdf]

MITF downregulation induces death in human mast cell leukemia cells and impairs IgE-dependent degranulation.

Elizabeth Proaño-Pérez , Laia Ollé , Yanru Guo , Cristina Aparicio, Mario Guerrero , Rosa Muñoz-Cano , Margarita Martin

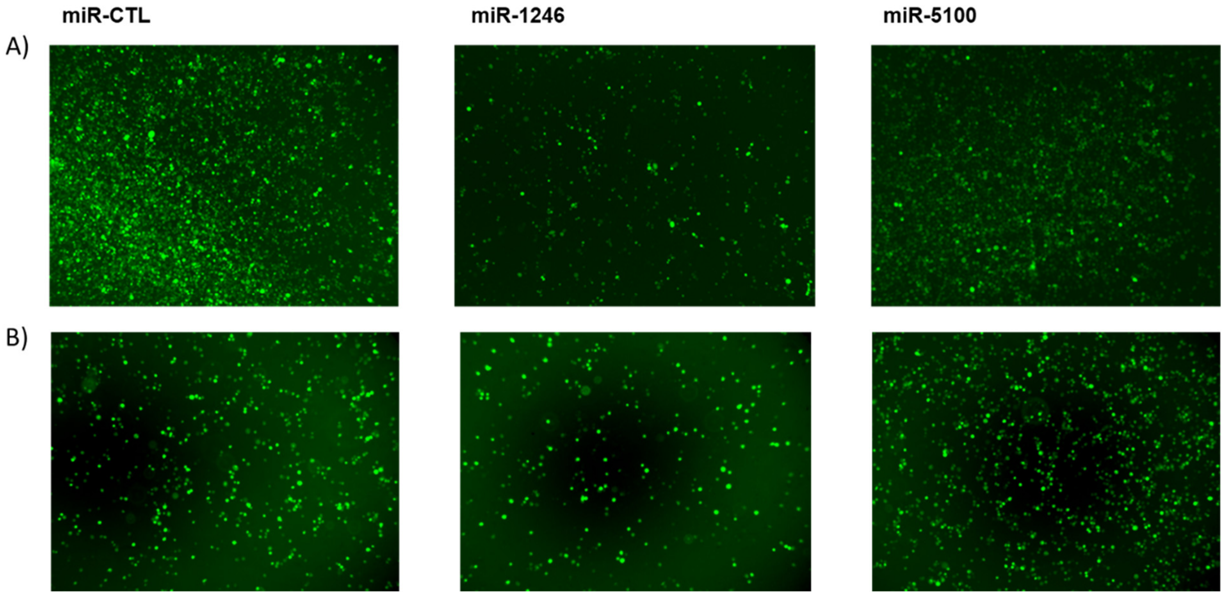

Figure S1. GFP-miRNAs expression in HMC-1 and LAD2. GFP expression was corroborated for control lentiviral transduction in both cell lines at 4th-day miR-CTL, miR-5100, and miR-1246 were measured by fluorescence microscopy (A) HMC-1 and (B) LAD2 cells.

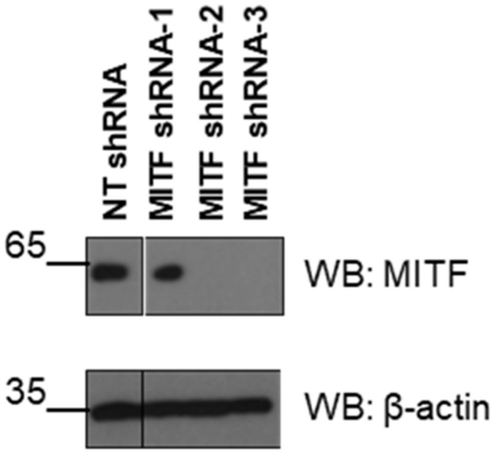

Figure S2. MITF silencing in HMC-1. Sequences MITF shRNA-1, MITF shRNA-2, and MITF shRNA-3 were validated in HMC-1 cells. Western blot shows MITF protein expression at 4th-day after lentiviral transduction.

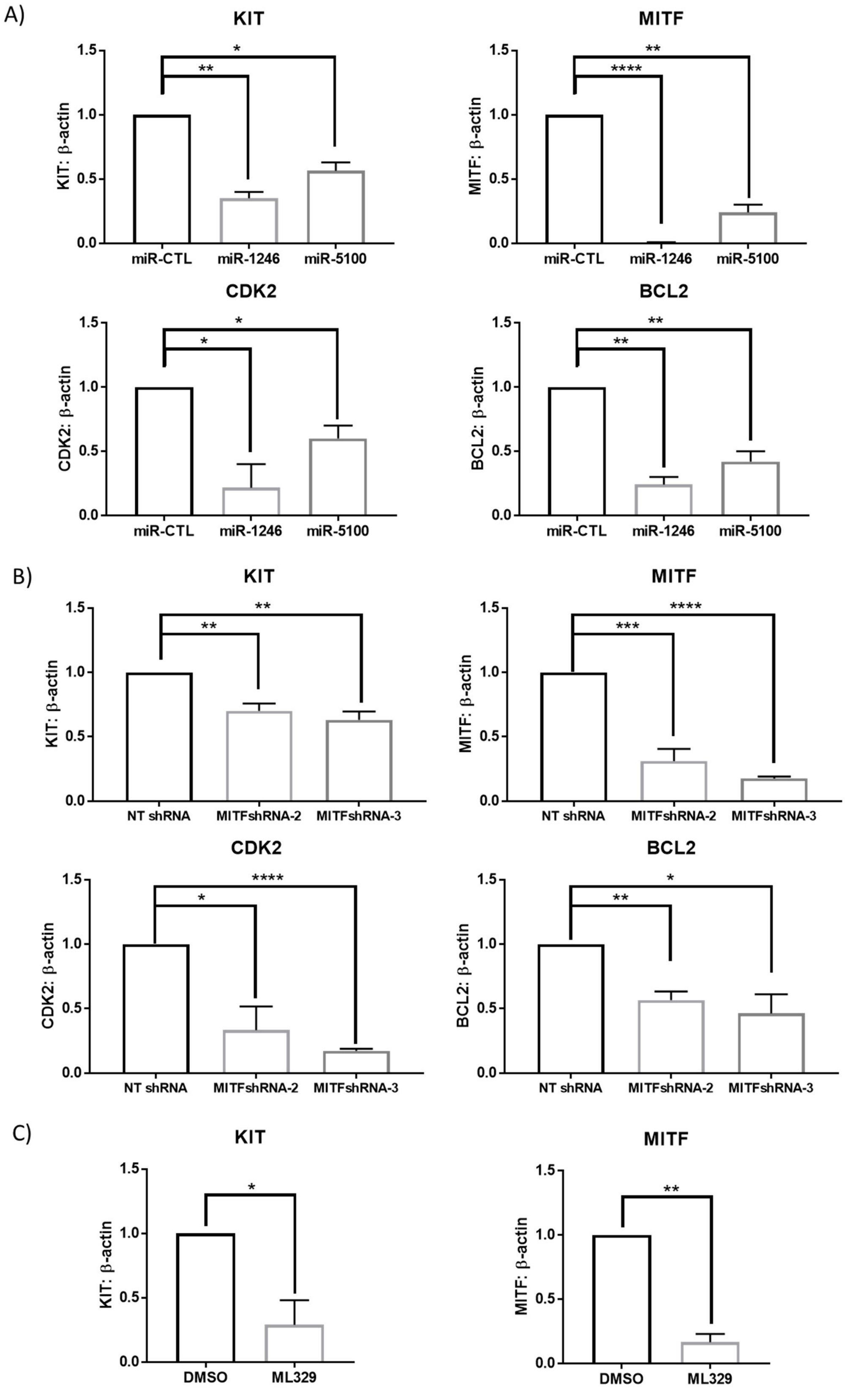

Figure S3. Band intensities from WB Figure 2A, 3A, and 4A. Graph depicting quantification of KIT: β-actin, MITF: β-actin, CDK2: β-actin and BCL2: β-actin ratios in the experimental set-up shown in Figure 2 (A); Figure 3 (B), Figure 4 (C). Unpaired T-test; mean ±SEM n = 3 independent experiments, (*p<0.05, **p< 0.01, ***p < 0.001, ****p 0.0001).
